# Prevalence and predictive factors of musculoskeletal injuries in triathletes: a cross-sectional study

**DOI:** 10.1186/s13102-025-01451-5

**Published:** 2025-12-09

**Authors:** Cassius Iury Anselmo-e-Silva, Aldair Darlan Santos-de-Araújo, Hygor Ferreira-Silva, Eliziane Oliveira da Silva, Isabelle Maria de Farias Brito, Camila de Lima Oliveira, Maria Julia Checo Melger, Caroline de Menezes Pinto, Fábio Sprada de Menezes, Natanael Teixeira Alves de Sousa

**Affiliations:** 1 Physical Therapy Department, Federal University of São Carlos, São Carlos, SP Brazil; 2 Department of Physical Therapy, Central University of Tiradentes, Maceió, AL Brazil; 3 Ironman Brazil Triathlon, Florianópolis, SC Brazil; 4 Cinemática Sport and Health, R. Olindina Campos Teixeira, Maceió, Al 57036-690 Brazil

**Keywords:** Musculoskeletal injury, Sport, Epidemiology, Triathlon

## Abstract

**Background:**

Triathlon combines swimming, cycling, and running over varying distances and has grown in popularity in recent years. Despite this, few studies investigate the prevalence and risk factors of injuries. This study aimed to analyze injury prevalence and distribution across body regions in Brazilian triathletes, and to identify predictive factors associated with injury development.

**Methodology:**

A cross-sectional study was conducted with 758 Brazilian triathletes through an online form. Data collected included sociodemographic and anthropometric information, sports participation duration, injuries in the past 12 months, and number of competitions. Descriptive statistics were applied, with group comparisons using t-test, Mann-Whitney, or Chi-square tests. Binary logistic regression identified injury predictors, with variables (*p* < 0.20) entered into multiple regression via forward selection. Model fit was assessed using R² Cox & Snell, R² Nagelkerke, and ROC curve analysis.

**Results:**

Of the 758 triathletes, 614 (81%) were male. More than half (*n* = 425) reported an injury in the past 12 months, most frequently in the lower limbs, particularly the knee (16.88%). Logistic regression revealed associations between injury occurrence and receiving prevention guidance from a physician (OR = 0.512; 95% CI: 0.320–0.818; *p* = 0.005), physiotherapist (OR = 0.249; 95% CI: 0.178–0.348; *p* < 0.001), and physical education professional (OR = 0.338; 95% CI: 0.244–0.467; *p* < 0.001). Participation in Ironman 70.3 distance in the last year increased injury likelihood (OR = 1.018; 95% CI: 1.216–2.339; *p* = 0.002). The ROC curve demonstrated good discriminative capacity (AUC = 0.759; 95% CI: 0.724–0.793; *p* = 0.001).

**Conclusion:**

Lower limb injuries were the most prevalent among Brazilian triathletes. Injury prevention guidance from physicians, physiotherapists, and physical education professionals was associated with reduced risk, whereas competing in Ironman 70.3 distance increased injury likelihood. The predictive model showed good accuracy.

**Supplementary Information:**

The online version contains supplementary material available at 10.1186/s13102-025-01451-5.

## Background

Triathlon is a multisport modality that combines swimming, cycling, and running in a continuous sequence of events, with athletes aiming to complete the course in the shortest time possible [1]. Requiring a unique combination of physical, mental, and endurance capabilities across each segment, triathlon is practiced in various formats, primarily differentiated by the distance covered in each event format [2, 3]. Among the available formats, the sprint, Olympic, half-Ironman (Ironman 70.3), and full Ironman distances are the most commonly contested worldwide [4, 5].

Undoubtedly, given the demand to maintain performance within a competitive timeframe in each modality, adequate energy balance, prior physical and psychological preparation, optimal cardiovascular conditioning, muscle quality, and ventilatory efficiency are essential prerequisites to meet the physiological demands elicited by each sport while sustaining oxygen consumption levels necessary to complete the race [6–8]. However, the combination of prolonged exertion and high-intensity effort in each segment of the triathlon exposes the body to a variety of injury mechanisms, including both acute and overuse injuries, even among athletes with a solid training background [9].

The risk of injury is directly influenced by the balance between an athlete’s physical capacity and the demands imposed by each specific sport modality [10, 11]. In runners, factors such as age, higher body mass, and a history of previous injuries are associated with an increased risk of musculoskeletal injuries [12–14]. In swimming, repetitive arm movements coupled with shoulder instability—though not exclusively—are known contributors to shoulder pain and constitute the most prevalent musculoskeletal injuries among competitive swimmers [15–17]. In cycling, increased weekly training or riding frequency, and interestingly, a history of chronic disease, have been associated with both acute traumatic injuries and those of gradual onset in both amateur and professional athletes [18].

The mechanisms underlying sports injuries are multifaceted and can be better understood through a complex systems approach, in which various components dynamically interact [19–21]. The interplay among biomechanical, physiological, psychological, and behavioral factors generates a web of unpredictable determinants that may lead to, and help explain, the onset of injuries [20, 22]. Nevertheless, advancing our understanding of the complexity of these systems and the interactions among their components holds promise for improving our grasp of injury mechanisms and for informing the development of strategic preventive interventions [23].

In summary, injury risk factors in triathletes depend on the nature of their mechanisms and can be broadly classified as intrinsic or extrinsic. Previous studies indicate that cyclists frequently experience overuse injuries, with the most commonly affected regions being the knees, lower back, cervical spine, wrists, and hands [24, 25]. In running, the most prevalent injuries include patellofemoral pain, medial tibial stress syndrome, plantar fasciitis, achilles tendinopathy, and stress fractures [26]. While shoulder injuries are common in swimming, they are not the only injuries reported [27, 28]. Other regions can be affected, such as the lower back, wrist and hand [29].

Although triathlon-related injuries are widely recognized in the literature, the number of studies specifically focused on risk factors and the most affected body regions has not yet been thoroughly explored. In this context, the primary aim of this study was to analyze the prevalence of injuries, considering their distribution by body region, in triathletes in Brazil. Secondarily, we aimed to explore and identify the factors associated with the development of injuries. We hypothesize that the prevalence of injuries across body regions is higher in areas more heavily involved in the specific training demands of swimming, cycling, and running, reflecting the repetitive movements associated with each modality.

## Methods

### Study design and ethical procedures

An online cross-sectional study was conducted in accordance with the Checklist for Reporting Results of Internet E-Surveys (CHERRIES) [30] and Strengthening the Reporting of Observational Studies in Epidemiology (STROBE statement) [31]. This study was approved by the Tiradentes University Center Human Ethics Committee (protocol number: 02704218.6.0000.5641) and was conducted in compliance with the Declaration of Helsinki. Data collection was carried out between January 2018 and November 2021. All participants signed an informed consent form before taking part in the study.

### Participants

An open-ended cross-sectional study was conducted through an online form applied to Brazilian triathletes, both remotely and in person during competitions. No minimum experience in the sport was required for participation.

Participants were recruited through announcements on social media, during competitions, and via outreach by the Brazilian Triathlon Confederation. A total of 758 volunteers (614 men and 144 women), aged between 27 and 49 years, participated in the study. A convenience sampling method was employed [32, 33].

Inclusion criteria for the study were: triathletes of both sexes, aged 18 years or older, who had participated in national triathlon competitions, regardless of race modality or category. Exclusion criteria for both sexes were: participants who did not meet the inclusion criteria and those who refused to complete the questionnaire.

### Procedures

Data were collected using a self-administered online form (Google Forms). The questions were developed by experienced physical therapists (NTAS, FSM and EOS). After the questionnaire was developed and reviewed, all authors approved the final version. To ensure clarity and understanding of the questions (written in Brazilian Portuguese), a pilot study was conducted prior to the start of data collection [34].

The questionnaire consisted of 24 questions, divided into the following sections: 1 – participant characteristics; 2 – sport practice: duration of sport practice, number of competitions in the last 12 months, number of swimming, cycling, and running training days per week; 3 – specific questions about injuries related to triathlon practice: “Have you sustained any musculoskeletal injuries in the last 12 months?”, location of injury occurrence. For data analysis, only injuries that led to athletes being removed from sports (time-loss) were considered [35, 36]; 4 – possible injury prevention measures: guidance on injury prevention from a physician, physical therapist, and physical education professional. Additionally, upon accessing the link, the participant was directed to the questionnaire’s homepage, where they could access the informed consent form, which described the study’s objectives and procedures. After agreeing to participate, the participant was directed to the questionnaire. Once the questionnaire was completed, an email was automatically sent to the principal investigator with the information provided by the participants.

Participants were asked about the duration of triathlon practice, the number of competitions in the last 12 months, and the number of training days per week to obtain quantitative responses. For the remaining questions, multiple-choice questions were used (response options: “yes or no”).

### Statistical analysis

Data were presented as mean ± standard deviation (SD), median and interquartile range (IQR25-75), or as absolute values and percentages (%), depending on the data distribution. The Kolmogorov-Smirnov test was used to assess the normality of continuous variables. For comparison between groups (men vs. women), the independent *t*-test was applied for normally distributed variables, while the Mann-Whitney *U* test was used when the assumption of normality was violated. Categorical variables were compared using the Chi-square test.

To identify predictors of injury occurrence among triathletes (yes or no), a simple binary logistic regression analysis was performed. Variables with a p-value < 0.20 in the univariate analysis were entered into a multiple logistic regression model using the forward selection method [37]. The final model was refined using the enter method to improve the explanation of variance (R^2^ Cox & Snell and R^2^ Nagelkerke). Results were reported as odds ratios (OR) with 95% confidence intervals (95% CI). To facilitate interpretation of the findings, a forest plot was constructed to graphically present the estimated effects of each variable. In the plot, OR values greater than 1 indicate a positive association with the outcome, while values less than 1 indicate a negative association. Confidence intervals that do not cross the reference value (OR = 1) suggest a statistically significant association. Multicollinearity was assessed using Variance Inflation Factor (VIF) and Tolerance statistics. All VIF values were below 5 and all Tolerance values were above 0.2, indicating the absence of multicollinearity among the independent variables [38]. The goodness-of-fit of the logistic regression model was assessed using the Hosmer–Lemeshow test, a p-value greater than 0.05 indicates no evidence of poor fit, suggesting that the model adequately fits the data [39].

Furthermore, Receiver Operating Characteristic (ROC) curve analysis was used to evaluate the discriminative power of age (years), practice time (years), Iroman 70.3 distance, guided by a Physician, physiotherapist and physical education professional on injury prevention in identifying musculoskeletal injuries [40]. Area under the curve (AUC) values were classified as follows: AUC < 0.5, low predictive capacity; 0.7 ≤ AUC < 0.8, good predictive capacity; and 0.8 ≤ AUC < 0.9, excellent predictive capacity. Cutoff points were established only when a good or excellent predictive capacity was observed (AUC ≥ 0.70). To assess the discriminative ability of the model, a binary logistic regression was performed, with injury occurrence in the past 12 months (0 = no; 1 = yes) as the dependent variable and age (years), practice time (years), Iroman 70.3, Guided by a Physician, physiotherapist and Physical Education Professional as independent variables. The predicted probabilities for each individual, derived from the logistic model, were used as a continuous variable to construct the ROC curve [41].

All analyses were conducted using the Statistical Package for the Social Sciences (SPSS) [IBM—SPSS, version 25.0 for Windows, Armonk, NY, <https://www.ibm.com/br-pt%3E/]. The forest plot was created using the GraphPad Prism version 8.0.1 for Windows (GraphPad Software, Boston, Massachusetts USA), < www.graphpad.com >. The probability of type 1 error occurrence was established at 5% for all tests (*p* < 0.05).

## Results

This study included 758 participants, predominantly male (81%) with an average age of 39 years. Regarding the anthropometric variables, men had significantly higher height (m), weight (kg), and BMI compared to women (*p* < 0.001). Additionally, men had a longer duration of sport practice than women (*p* = 0.007). No significant statistical difference was observed between the sexes regarding the number of reported injuries (56.06% in men and 56.25% in women, *p* = 0.961) (Table 1).

**Table 1:** Participant characteristics

Variables	All volunteers (*n* = 758)	Men (*n* = 614)	Women (*n* = 144)	*P* value
Age (years)	39 (32–45)	39 ± 10	38 ± 11	0.265
18–24	43 (5.67)	32 (5.21)	11 (7.64)	0.251
25–29	72 (9.50)	55 (8.96)	17 (11.81)	
30–34	129 (17.02)	102 (16.61)	27 (18.75)	
35–39	153 (20.18)	129 (21.01)	24 (16.67)	
40–44	159 (20.98)	133 (21.66)	26 (18.06)	
45–49	85 (11.21)	71 (11.56)	14 (9.72)	
50–54	62 (8.18)	44 (7.17)	18 (12.50)	
55–59	34 (4.49)	30 (4.89)	4 (2.78)	
≥ 60	21 (2.77)	18 (2.93)	3 (2.08)	
Weight (Kg)	72.50 (65–80)	75.59 ± 10.37	59.99 ± 8.93	< 0.001*
Height (m)	1.74 (1.69–1.79)	1.75 ± 0.13	1.64 ± 0.06	< 0.001*
BMI (Kg/m²)	23.71 (22.15–25.21)	24.53 ± 7.15	22.19 ± 2.98	< 0.001*
Underweight	16 (2.11)	8 (1.30)	8 (5.55)	< 0.001*
Eutrophic	528 (69.66)	410 (66.77)	118 (81.94)	
Overweight	189 (24.93)	176 (28.66)	13 (9.02)	
Obesity I	22 (2.90)	17 (2.77)	5 (3.47)	
Obesity II	3 (0.40)	3 (0.49)	0 (0)	
Obesity III	0 (0)	0 (0)	0 (0)	
Practice time (years)	3 (2–6)	4 (2–6)	3 (1–5)	0.007*
Modalities (last 12 months)				
Sprint-distance	421 (55.54)	330 (53.75)	91 (63.19)	0.040*
Olympic-distance	307 (40.50)	248 (40.39)	59 (40.97)	0.898
Ironman 70.3 distance	354 (46.70)	304 (49.51)	50 (34.72)	< 0.001*
Ironman full-distance	98 (12.93)	83 (13.52)	15 (10.41)	0.318
Injury				
Yes	425 (56.06)	344 (56.02)	81 (56.25)	0.961
No	333 (43.93)	270 (43.97)	63 (43.75)	
Injury during training	355 (46.83)	289 (47.06)	66 (45.83)	0.266
Injury during competition	35 (8.62)	28 (4.56)	7 (4.86)	
Injury in both (training and competition)	41 (5.40)	29 (4.72)	12 (8.33)	
Number of competitions (12 months)	7 (6–7)	6.00 ± 1.00	6.00 ± 1.00	0.220
Cycling training (days/week)	3 (3–4)	3.00 ± 1.00	3.00 ± 1.00	0.380
Swimming training (days/week)	2 (2–3)	2.00 ± 1.00	2.00 ± 1.00	0.686
Running training (days/week)	3 (3–4)	3.00 ± 1.00	3.00 ± 1.00	0.606
Professional guidance on injury prevention				
Physician	134 (17.67)	105 (17.10)	29 (20.13)	0.390
Physiotherapist	337 (44.45)	269 (43.81)	68 (47.22)	0.458
Physical Education Professional	368 (48.54)	286 (46.58)	82 (56.94)	0.025*

Data in mean ± standard deviation, median and interquartile range (IQR25-75) or absolute value and percentage (%)

*Kg* kilos, *m* meter,* BMI* Body mass index, *Kg/m²* kilogram per square metre

*Statistical significance for comparison between groups (*p* < 0.05)

### Injury distribution by body region

In the total sample, the areas with the highest self-reported injury rates were: the knee (16.88%), lower leg (16.49%), and hip (9.76%). Regarding sex, it was observed that women reported a significantly higher proportion of hip joint injuries (16.66%) compared to men (8.14%; *p* = 0.002). For the other self-reported injuries, no statistically significant differences were observed (*p* > 0.05). (Table 2).

**Table 2:** Distribution of injuries by body region in triathletes

Body region	All volunteers (*n* = 758)	Men (*n* = 614)	Women (*n* = 144)	*P* value
Cervical	8 (1.05)	5 (0.81)	3 (2.08)	0.180
Thoracic	4 (0.52)	3 (0.48)	1 (0.69)	0.759
Lumbar	41 (5.40)	31 (5.04)	10 (6.94)	0.365
Chest	2 (0.26)	1 (0.16)	1 (0.69)	0.263
Shoulder	72 (9.49)	61 (9.93)	11 (7.63)	0.398
Arm	5 (0.66)	5 (0.81)	0 (0)	0.277
Elbow	3 (0.39)	3 (0.48)	0 (0)	0.401
Forearm	2 (0.26)	2 (0.326)	0 (0)	0.493
Wrist	8 (1.05)	8 (1.30)	0 (0)	0.169
Hands	3 (0.39)	3 (0.48)	0 (0)	0.401
Fingers	2 (0.26)	2 (0.32)	0 (0)	0.493
Hip	74 (9.76)	50 (8.14)	24 (16.66)	0.002*
Thigh	72 (9.49)	55 (8.95)	17 (11.80)	0.294
Knee	128 (16.88)	101 (16.45)	27 (18.75)	0.507
Leg	125 (16.49)	105 (17.10)	20 (13.88)	0.350
Ankle	55 (7.25)	42 (6.84)	13 (9.02)	0.362
Foot	85 (11.21)	67 (10.91)	18 (12.50)	0.587
Toes	7 (0.92)	4 (0.65)	3 (2.08)	0.106

Data in absolute value and percentage (%)

*Statistical significance for comparison between groups (*p* < 0.05)

### Risk factors associated with the occurrence of injuries

Table 3 displays the different models of simple binary logistic regression potentially associated with the risk of injury in triathletes. A statistically significant association was observed between age (β = 0.018; OR = 1.018; *p* = 0.020), duration of practice in the sport (β = 0.031; OR: 1.031; *p* = 0.032), participation in Ironman 70.3 events (β = 0.334; OR: 1.396; *p* = 0.023), prior guidance from a physician (β = −0.995; OR: 0.370; *p* < 0.001), prior guidance from a physical therapist (β = −1.371; OR: 0.254; *p* < 0.001), and prior guidance from a physical education professional (β = −1.118; OR: 0.327; *p* < 0.001).

**Table 3:** Binary logistic regression analysis of factors potentially associated with injuries in triathletes

VARIABLES	β	S.E.	Wald	*P* value	Odds ratio (CI95%)	*R*^2^ Cox & Snell	*R*^2^ Nagelkerke
Age (years)	0.018	0.008	5.388	0.020*	1.018 (1.003, 1.033)	0.007	0.010
Weight (kg)	0.003	0.006	0.197	0.657	1.003 (0.991, 1.015)	0.000	0.000
Height (m)	1.085	0.633	2.212	0.086	2.960 (0.856, 10.239)	0.004	0.006
BMI (kg/m²)	−0.009	0.012	0.610	0.435	0.991 (0.969, 1.014)	0.001	0.001
Practice time (years)	0.031	0.014	4.625	0.032*	1.031 (1.003, 1.060)	0.006	0.009
Last 12 months (0, no; 1, yes)							
Sprint-distance	0.023	0.147	0.024	0.877	1.023 (0.766, 1.365)	0.000	0.000
Olympic-distance	−0.198	0.150	1.747	0.186	0.821 (0.612, 1.100)	0.002	0.003
Ironman 70.3 distance	0.334	0.147	5.146	0.023*†	1.396 (1.046, 1.862)	0.007	0.009
Ironman full-distance	0.328	0.217	2.283	0.131	1.388 (0.907, 2.123)	0.003	0.004
Number of competitions	0.105	0.085	1.536	0.215	1.111 (0.941, 1.312)	0.002	0.003
Guidance on injury prevention (0, no; 1, yes)							
Physician	−0.995	0.214	21.619	< 0.001*†	0.370 (0.243, 0.562)	0.031	0.042
Physiotherapist	−1.371	0.159	74.746	< 0.001*†	0.254 (0.186, 0.346)	0.101	0.135
Physical Education Professional	−1.118	0.153	53.539	< 0.001*†	0.327 (0.242, 0.441)	0.071	0.095

*β* Beta, *kg* kilos, *m* meter, *BMI* Body mass index, *S.E*. standard error, *%* percentage, *C.I*. confidence interval

*Statistical significance for binary logistic regression analysis (*p* < 0.05)

†Statistical significance for multiple regression analysis (*p* < 0.020)

### Multiple logistic regression

**Fig. 1:**
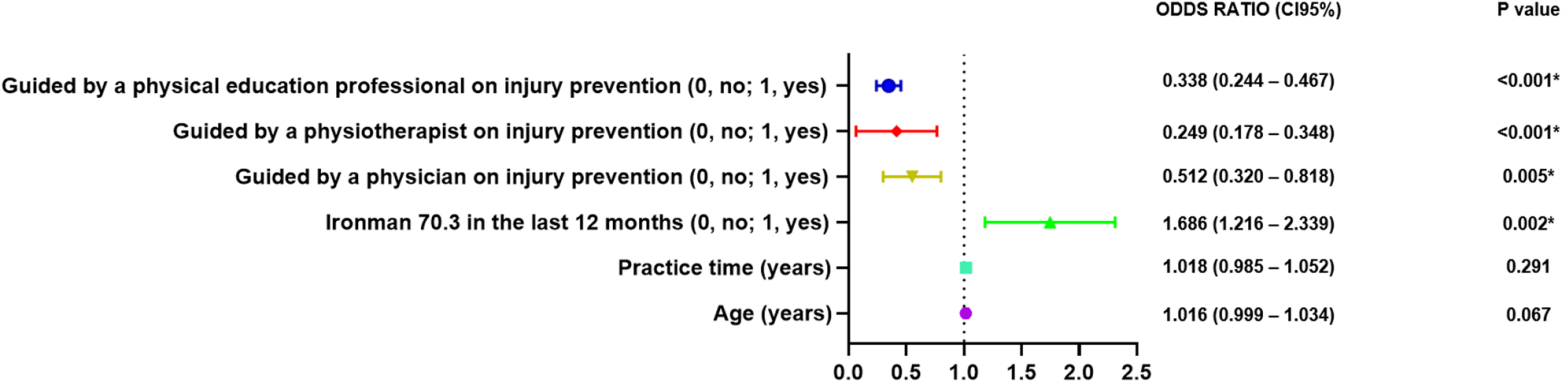
Forest plot from the multivariable logistic regression model predicting injuries in triathletes. CI: confidence interval; %: percentage. Statistical significance for logistic regression analysis (p<0.05)

The multiple logistic regression analysis was performed to identify factors associated with the occurrence of injuries in triathletes (Table 4; Fig. 1). The prior guidance from Physician had a β coefficient of −0.670 (*p* = 0.005), with an OR of 0.512 (95% CI: 0.320–0.818). This indicates that those who received guidance from a Physician have a significantly lower probability of injury, with a 49% reduced chance compared to those who did not receive guidance. Additionally, prior guidance from a physical therapist had a β coefficient of −1.392 (*p* < 0.001), with an OR of 0.249 (95% CI: 0.178–0.348). This indicates that those who received guidance from a physical therapist have a significantly lower probability of injury, with a 75.1% reduced chance compared to those who did not receive guidance.

**Table 4:** Multiple logistic regression analysis of factors potentially associated with injuries in triathletes

VARIABLES	β	S.E.	Wald	*P* value	Odds ratio (CI95%)	*R*^2^ Cox & Snell	*R*^2^ Nagelkerke	Hosmer and Lemeshow
Constant	1.180	0.427	7.644	0.006*		0.187	0.251	0.097
Age (years)	0.016	0.009	3.357	0.067	1.016 (0.999, 1.034)			
Practice time (years)	0.018	0.017	1.115	0.291	1.018 (0.985, 1,052)			
Iroman 70.3 distance in the last 12 months (0, no; 1, yes)	0.523	0.167	9.806	0.002*	1.686 (1,216, 2,339)			
Guided by a Physician on injury prevention (0, no; 1, yes)	−0.670	0.239	7.833	0.005*	0.512 (0.320, 0.818)			
Guided by a physiotherapist on injury prevention (0, no; 1, yes)	−1.392	0.172	65.816	< 0.001*	0.249 (0.178, 0.348)			
Guided by a Physical Education Professional on injury prevention (0, no; 1, yes)	−1.085	0.165	43.127	< 0.001*	0.338 (0.244, 0467)			

*β* Beta, *S.E*. standard error

*Statistical significance for logistic regression analysis (p < 0.05)

Similarly, prior guidance from physical education professional had a β coefficient of −1.085 (*p* < 0.001), with an OR of 0.338 (95% CI: 0.244–0.467). This indicates that those who received guidance from a Physical Education Professional have a significantly lower probability of injury, with a 66.2% reduced chance compared to those who did not receive guidance.

Moreover, participation in Ironman 70.3 distance events showed a β coefficient of 0.523 (*p* = 0.002) and an OR of 1.686 (95% CI: 1.216–2.339). This indicates that triathletes who participate in Ironman 70.3 distance have 68.6% higher odds of sustaining an injury compared to those who do not. The age (*p* = 0.067) and time of practice (*p* = 0.291) did not reach statistical significance. The final model showed a low explanatory power (Cox & Snell R² = 0.187; Nagelkerke R² = 0.251), indicating that only 18–25% of the variance was explained. Furthermore, all variables included in the final multiple logistic regression model showed no evidence of multicollinearity, as all VIF values were < 5 and all Tolerance values were > 0.2 [38].

### ROC curve and Hosmer–Lemeshow test

**Fig. 2:**
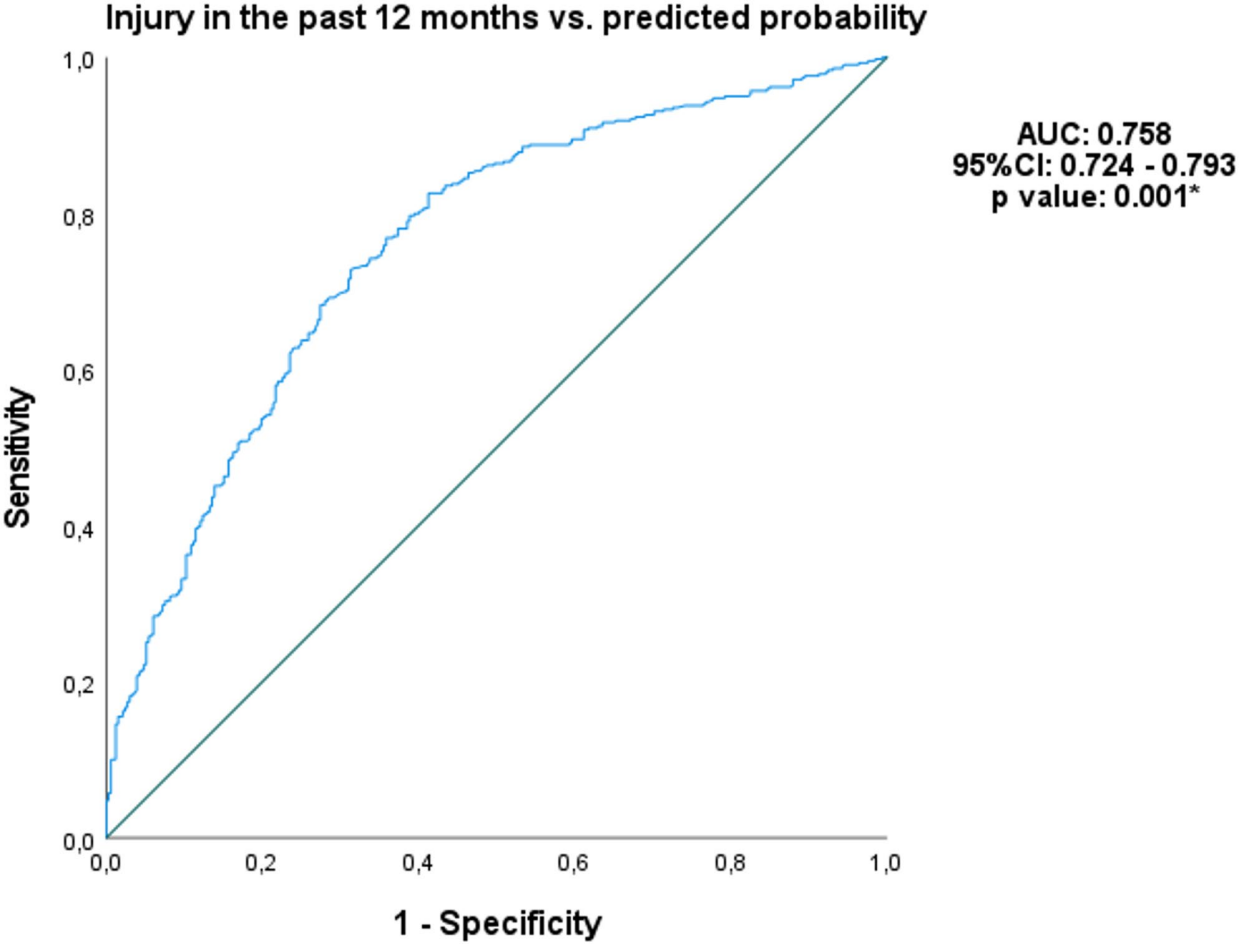
ROC curve for the prediction of injury in the past 12 months from the logistic model. AUC: area under curve; %: percentage; CI: confidence interval. *The AUC was significantly different from 0.05

According to the ROC curve analysis (Fig. 2), the logistic regression model showed a significant and good discriminative capacity (AUC = 0.759, 95% CI: 0.724–0.793; *p* = 0.001). The model included the following predictor variables: age, practice time (years), participation in Ironman 70.3, and guidance from a Physician, Physiotherapist, or Physical Education Professional. Furthermore, the model demonstrated good fit and calibration, as indicated by the Hosmer–Lemeshow test (*p* = 0.097).

## Discussion

The objective of this study was to analyze the prevalence of injuries in triathletes in Brazil, considering the distribution of injuries by body region, as well as identifying potential associations. The main findings were: (1) the knee, leg, and foot regions account for the highest proportion of injury prevalence in Brazilian triathletes; (2) prior guidance regarding injury risk provided by a physician, physical therapist and physical education professional was associated with a significant reduction in the probability of injuries; and (3) participation in ironman 70.3 is associated with a higher risk of injuries; and (4) logistic regression model showed a significant and good discriminative capacity.

### Injury prevalence

The prevalence of injuries was similar between male and female triathletes, with the notable exception of the hip region, where women exhibited a significantly higher proportion of injuries (16.66%) compared to men (8.14%). This result is particularly interesting considering that a recent systematic review identified the male sex as a key determinant for the development of hip injuries in all sports [42]. This discrepancy may reflect sport-specific demands or underline the importance of analyzing sex-based differences in injury patterns within each athletic population. Similar findings have been reported in previous studies, which also observed a higher number of male participants [27, 43, 44]. We believe that sex differences in triathlon participation may vary according to regional or geographical factors.

In recent years, the growing interest in understanding sex and gender-based differences in sports has contributed to the development of more individualized and targeted prevention strategies. It is now well established that men and women differ not only in anatomical structure but also in neuromuscular control and biomechanical responses during dynamic tasks [45, 46]. For instance, women have been shown to exhibit increased knee adduction and internal rotation, reduced hip and knee flexion angles, increased dynamic knee valgus, increased quadriceps activity, and decreased hamstring activity [46, 47]. These characteristics may increase joint stress and could partially explain the greater vulnerability of the hip region in female triathletes in our sample.

As mentioned earlier, anatomical differences between them influence performance in dynamic activities. These differences may include variations in muscle mass, strength, body composition, and kinematic variables which are crucial for balance and stability [48–50].

The knee region was the most affected among the athletes, a finding that corroborates the results of Minghelli et al. [51]. However, this result differs from the study by Bertola et al. [52], which identified the calf as the most affected area. In our study, the lower leg (16.49%) and foot (11.21%) also showed high injury prevalence, corroborating the findings of Gosling et al. [53] and Crunkhorn et al. [54], who reported high rates of injury in distal lower limb regions among triathletes. On the other hand, V. E. Vleck et al. [4] identified the lumbar spine and knee as the most frequently affected areas, highlighting how differences in sample characteristics, competition levels, and training volumes may influence injury profiles.

The predominance of lower limb injuries is not unexpected given the cumulative mechanical load and repetitive stress imposed by triathlon modalities, particularly running and cycling. These activities are characterized by high volumes of training and a predominance of cyclical movements that place repeated stress on the joints, tendons, and muscles of the lower extremities [50]. In particular, the running phase has been consistently associated with the highest injury rates in triathlon, especially due to impact loading and insufficient recovery between training sessions [28]. The high incidence of knee, leg, and hip injuries observed in our sample indicates that these body segments deserve greater attention in future analyses and discussions. Further studies are warranted to evaluate the effectiveness of injury prevention interventions specifically aimed at these anatomical regions, particularly within the triathlon context.

One methodological constraint of the present study was the absence of a standardized definition of musculoskeletal injury provided to participants. Without a clear operational definition, responses may have reflected individual perceptions of injury, ranging from transient discomfort to conditions requiring medical intervention or training cessation. This subjective interpretation could have influenced the accuracy of self-reported prevalence rates, potentially leading to over- or underestimation of injury occurrence. Future studies should adopt and explicitly report standardized injury definitions, such as those proposed by international consensus statements, to enhance the consistency and comparability of findings in triathlon injury research [55, 56].

### Risk factors

Interestingly, our results revealed that age is not associated with the occurrence of injuries. A recent systematic review [28] on short-distance triathletes highlighted that age appears to influence injury occurrence in this population. However, there is no consensus in the literature on whether this variable is associated with the occurrence of injury in different sports modalities, and specifically in triathlon, the results remain paradoxical. This paradoxical effect can be observed in two studies involving triathletes. One reported that age ≥ 40 years was associated with a higher risk of injury [51], while another found no statistically significant difference in injury incidence rates between groups aged above and below 40 [57]. Additionally, age was not identified as a risk factor for injury incidence due to overuse in a sample of 131 triathletes [58]. Furthermore, among recreational runners, age was found to be a risk factor for injury in younger men but not in women [59]. These inconsistencies may reflect the heterogeneity of triathlon populations and the multifactorial nature of injuries. In our study, Age is not associated with the occurrence of injuries. This lack of associations may be related to the predominance of older and potentially more experienced athletes (median age = 39 years), who are likely to have better training management and greater adherence to preventive behaviors.

Another point is that receiving prior guidance from a physician, physiotherapist, or physical education professional regarding injury prevention was significantly associated with a lower likelihood of sustaining injuries, with a reduced chance of 49%, 75.1%, and 66.2%, respectively, compared to those who did not receive such guidance. To the best of our knowledge, this is the first time that injury-related guidance and its potential protective role have been explored and statistically associated with a reduced risk of injury in triathletes using robust statistical analysis. Previously, one study noted that the presence of a training coach or access to medical care did not appear to be associated with injury development [60]. Interestingly, more than half of the participants (55.1%) sought medical care more than once per year. However, the authors inferred this finding without applying appropriate statistical methods to evaluate the association [60]. Thus, it seems plausible to believe that prior guidance from a healthcare professional on injury prevention may exert a beneficial effect on the outcomes observed in this study. This is supported by the notion that health education, particularly when it addresses contextual factors can raise awareness of risks, promote preventive behaviors, and consequently reduce the incidence of injuries over time [61]. This has also been observed in individuals with chronic pain [62, 63], such as those affected by patellofemoral pain [64], low back pain [65, 66], and neck pain [67]. In these populations, educational programs and interventions guided by health professionals may be shown to improve adherence to preventive strategies, reduce pain, and improve functional outcomes. This reinforces the importance of multidisciplinary engagement in athlete preparation and the potential impact of structured education on musculoskeletal injuries. However, it is important to acknowledge that the observed association between guidance from physicians, physiotherapists, and physical education professionals regarding injury prevention and the lower occurrence of musculoskeletal injuries may be partially explained by unmeasured confounding factors, such as the athlete’s previous experience, socioeconomic status, or general preventive attitudes.

Conversely, participation in ironman 70.3 distance was associated with a significantly increased risk of injury, with triathletes in this category showing a 68.6% higher chance of injury compared to those who do not engage in ironman 70.3 distance. This finding is supported by Minghelli et al. [51], who also reported a higher incidence of injuries among long-distance athletes. Similarly, Andersen et al. [44] indicated that long-distance triathletes commonly experience overuse injuries, which represent the majority of injury cases in this category of athletes. The elevated training volume required for these competitions, coupled with prolonged mechanical load and the cumulative effect of fatigue over time, seems to create a scenario particularly conducive to overuse injuries. These findings underline the importance of tailored load management and recovery strategies to mitigate injury risk in these athletics.

It is important to acknowledge that the retrospective design of this study increases the risk of recall bias, which may have affected the accuracy of the self-reported injuries and reduced the reliability of some associations observed. Additionally, the injury registration form was not a standardized or validated tool, which limits the validity of the measurements. This limitation should be considered when interpreting the findings, as participants may have under- or over-reported previous injuries or training exposures. Nonetheless, the consistency of our results with previous literature supports their overall plausibility.

Although caution is required due to potential recall bias, the discriminative capacity demonstrated by ROC curve analysis may contribute to strengthening confidence in the observed associations. ROC curve analysis revealed significant associations between injury occurrence in the past 12 months and logistic model. These associations demonstrated good discriminative capacity of the analyzed variables, with AUC values above 0.70. To our knowledge, no previous study has evaluated such predictive performance in triathletes. Our findings also suggest that additional factors, not captured in our comprehensive assessment, likely contribute to injury risk, underscoring the multifactorial nature of musculoskeletal injuries [21] in this population. Future research should explore other associative factors, such as biomechanical, ergonomic, socioeconomic, and psychosocial variables, to refine risk stratification strategies.

### Clinical impact

The findings of this study have relevant clinical implications for understanding injury prevalence in triathletes. First, athletes who received prior guidance from physiotherapists, physicians, or physical education professionals showed lower injury rates, highlighting the potential association between individualized education, preventive counseling, and reduced injury risk. To confirm the associations of these interventions on injuries in triathletes, new clinical trials that incorporate structured educational strategies into training routines. Second, the increased injury risk observed in athletes competing in Iroman 70.3 distance reinforces the need for careful load management and recovery planning. These athletes are exposed to higher cumulative mechanical stress and fatigue, which may predispose them to overuse injuries. Coaches and healthcare professionals should consider periodized training programs that balance performance development with adequate recovery. Finally, the observed differences in injury distribution between the sexes, particularly the higher prevalence of hip injuries in women, reinforce the importance of personalized approaches to prevention, considering the athlete’s individuality. Future clinical trials should test the effects of strength training routines, especially focusing on the lower limbs and hip stability, on injury risk and improving overall performance in triathletes.

### Limitations

Although our study included a substantial sample size, the findings should be interpreted with caution due to several limitations: (1) the cross-sectional design precludes causal inferences; (2) our results are limited to the Brazilian triathlete population; (3) The data were collected using a self-report form referring to the previous 12 months, which was not standardized or previously validated, potentially introducing recall bias and limiting the validity of the measurements; (4) The retrospective design and self-reported nature of the study introduce a risk of recall bias, which may have affected the accuracy of the reported information on injuries and exposures; (5) The collection period was broad (2018–2021), covering the time of the COVID-19 pandemic. This fact may have led to changes in the athletes’ training conditions, such as a reduction in training volume, which could impact our results; and (6) The adopted injury definition included only cases that resulted in time-loss from sports participation. As highlighted by van Mechelen et al. [68], restricting injury definitions to those requiring medical treatment or resulting in absence tends to cover mainly acute and severe cases, ignoring the “tip of the iceberg” of overuse injuries that may not cause withdrawal but lead to reductions in training volume or intensity. In endurance sports such as triathlon, where gradual overload is common, adopting a broader definition of “substantial injury”, considering reductions in performance or training load can provide a more comprehensive picture of the injury burden.

## Conclusion

The lower limbs are the most frequently injured segments among Brazilian triathletes, particularly the knees, legs, and feet. Furthermore, our logistic regression model demonstrated that physician, physical therapist and physical education professional guidance and injury prevention was associated with lower injury odds in this population. In contrast, participation in Ironman 70.3 distance in the past year was associated with an increased risk of injury. The model accounts for approximately 18% to 25% of the variance in the dependent variable.

## Supplementary Information

Supplementary Material 1

## Data Availability

Authors will share the dataset upon request.

## Abbreviations

AUC: Area under the curve
CHERRIES: Checklist for Reporting Results of Internet E-Surveys
STROBE: Strengthening the Reporting of Observational Studies in Epidemiology
SD: Standard deviation
IQR25-75: IQR25-75
%: Percentage
OR: Odds ratios
95% CI: 95% Confidence Interval
m: Meters
Kg: Kilogram
β: Beta
Roc Curve: Receiver Operating Characteristic Curve
VIF: Variance Inflation Factor
