# Supplementary material for: Prevalence and predictive factors of musculoskeletal injuries in triathletes: a cross-sectional study
Source: BMC Sports Sci Med Rehabil. 2025 Dec 9;18:20. doi: 10.1186/s13102-025-01451-5 (PMC12802025; doi:10.1186/s13102-025-01451-5)
Supplement: Supplementary file 1 — Supplementary Material 1 [file 13102_2025_1451_MOESM1_ESM.pdf]

ASSESSMENT FORM

Personal Information

Name: ______________________________ Date of assessment: ________________________

Contact Number:

Email:

City:

State:

Age:

Date of Birth:

Height:

Weight:

Sex:

- ☐ Male
- ☐ Female

Sports Practice

How long have you been practicing triathlon?

How many triathlon competitions have you participated in over the last 12 months?

Did you participate in the sprint distance in the last 12 months?

- ☐ Yes
- ☐ No

Did you participate in the Olympic distance in the last 12 months?

- ☐ Yes
- ☐ No

Did you participate in the Ironman 70.3 distance in the last 12 months?

- ☐ Yes
- ☐ No

Did you participate in the Ironman full distance in the last 12 months?

- ☐ Yes
- ☐ No

Swimming training

How many days per week do you train swimming?

What is your average swimming training time per day?

- ☐ 30 minutes
- ☐ 1 hour
- ☐ 1 hour 30 minutes
- ☐ 2 hours
- ☐ 2 hours 30 minutes
- ☐ 3 hours
- ☐ 3 hours 30 minutes
- ☐ 4 hours

Cycling training

How many days per week do you train cycling?

What is your average cycling training time per day?

- ☐ 30 minutes
- ☐ 1 hour
- ☐ 1 hour 30 minutes
- ☐ 2 hours
- ☐ 2 hours 30 minutes
- ☐ 3 hours
- ☐ 3 hours 30 minutes
- ☐ 4 hours

Running training

How many days per week do you train running?

What is your average running training time per day?

- ☐ 30 minutes
- ☐ 1 hour
- ☐ 1 hour 30 minutes
- ☐ 2 hours
- ☐ 2 hours 30 minutes
- ☐ 3 hours

- ☐ 3 hours 30 minutes
- ☐ 4 hours

Injuries Related to Triathlon Practice

For the purposes of this survey form, consider an ***injury*** any physical damage that prevents full participation in all planned activities and training for a specific day, for a period exceeding 24 hours, from midnight until the end of the day the injury was sustained [1–3].

Have you had any musculoskeletal injury in the last 12 months?

- ☐ Yes
- ☐ No

Did your injury occur during training, competition, or both?

- ☐ Training
- ☐ Competition
- ☐ Both

If you answered yes to musculoskeletal injury in the last 12 months, which body region was affected?

- ☐ Cervical
- ☐ Thoracic
- ☐ Lumbar
- ☐ Chest
- ☐ Shoulder
- ☐ Arm
- ☐ Elbow
- ☐ Forearm
- ☐ Wrist
- ☐ Hands
- ☐ Fingers
- ☐ Hip
- ☐ Thigh
- ☐ Knee
- ☐ Leg
- ☐ Ankle
- ☐ Foot
- ☐ Toes

Professional Guidance on Injury Prevention

Have you received guidance on injury prevention from a Physician?

- ☐ Yes
- ☐ No

Have you received guidance on injury prevention from a Physiotherapist?

- ☐ Yes
- ☐ No

Have you received guidance on injury prevention from a Physical Education Professional?

- ☐ Yes
- ☐ No

1. Allen N, Nevill A, Brooks J, Koutedakis Y, Wyon M. Ballet injuries: injury incidence and severity over 1 year. J Orthop Sports Phys Ther. 2012;42:781–90. https://doi.org/10.2519/JOSPT.2012.3893.

2. López-Valenciano A, Raya-González J, Garcia-Gómez JA, Aparicio-Sarmiento A, Sainz de Baranda P, De Ste Croix M, et al. Injury Profile in Women's Football: A Systematic Review and Meta-Analysis. Sports Med. 2021;51:423–42. https://doi.org/10.1007/S40279-020-01401-W.

3. Brooks JHM, Fuller CW, Kemp SPT, Reddin DB. A prospective study of injuries and training amongst the England 2003 Rugby World Cup squad. Br J Sports Med. 2005;39:288–93. https://doi.org/10.1136/BJSM.2004.013391.
